# How should multiple myeloma research change in a patient-oriented world? Findings and lessons from the pan-Canadian myeloma priority setting partnership

**DOI:** 10.1186/s40900-023-00476-9

**Published:** 2023-07-29

**Authors:** Sarah Bridges, Samantha Fowler, Lauren McLaughlin, Marc Robichaud, Barbara Ridgway, Donna Reece, Kevin Song, Lorelei Dalrymple, Robin Sully, Sharon Nason, Suzanne Rowland, Trish MacDonald, William Paine, Adrienne Gulliver, Anthony Reiman

**Affiliations:** 1 Office of Research Services, Horizon Health Network, Saint John, NB Canada; 2 Maritime SPOR SUPPORT Unit, Halifax, NS Canada; 3 Vitalité Health Network, Moncton, NB Canada; 4 Canadian Myeloma Priority Setting Partnership Steering Group, Doval, QC Canada; 5 Clinical Research Unit, Princess Margaret Cancer Centre, Toronto, ON Canada; 6 Leukemia/Bone Marrow Transplant Program, Vancouver General Hospital, Vancouver, BC Canada; 7 Department of Oncology, Saint John Regional Hospital, Horizon Health Network, 400 University Avenue, Saint John, NB E2L 4L2 Canada; 8 Department of Biological Sciences, University of New Brunswick, Saint John, NB Canada; 9 Department of Medicine, Dalhousie University, Saint John, NB Canada

**Keywords:** Priority setting, Patient and public involvement, Patient engagement, Multiple myeloma

## Abstract

**Background:**

Over the last decade there has been considerable research into the treatment, management, and quality of life of people living with multiple myeloma. However, there has been limited investigation into topics deemed important to patients and caregivers within this community. We conducted a James Lind Alliance Priority Setting Partnership to establish the ‘Top 10 Priorities for Myeloma Research’, informed by patient and public partners.

**Methods:**

A research team and steering group were established in 2019 to conduct the myeloma priority setting partnership. Steering group members included patients, caregivers, and healthcare providers who advised the research team and oversaw the scope of the project, grounded on their lived experience. Following the James Lind Alliance guidelines for identification and ranking of research questions, we used surveys and a virtual workshop to collect and prioritize questions posed by myeloma patients, caregivers, and healthcare providers across Canada.

**Results:**

The Top 10 list of priorities for myeloma research was finalized at the consensus-building workshop and encompassed questions related to diagnosis, treatment, management, and living well with myeloma. A final participant evaluation survey elicited a positive response.

**Interpretation:**

The myeloma priority setting partnership identified the research priorities of people living with myeloma, caregivers, and healthcare providers to inform clinical research on this disease going forward. This project underscores the importance of patient and public engagement in the identification of research questions, highlighting the concerns of people affected by myeloma to ultimately improve the lives of people living with this disease.

**Supplementary Information:**

The online version contains supplementary material available at 10.1186/s40900-023-00476-9.

## Abstract (plain language summary)

Research on multiple myeloma, a rare blood cancer, rarely focuses on topics that are personally important to people living with myeloma, their caregivers, and their healthcare providers. The purpose of this study was to complete a priority setting partnership, following guidelines from the James Lind Alliance, to identify unanswered research questions from people directly affected by myeloma. The project was guided by a steering group of people living with myeloma, their caregivers, and healthcare providers who informed all stages of this process including two questionnaires to collect and rank questions and a priority setting workshop. The workshop brought together representatives from each group to form the final ‘Top 10 Priorities for Myeloma Research’, which included questions related to diagnosis, treatment, management, and living well with myeloma. This process allowed us to identify the research priorities of the myeloma community and highlighted the importance of including patients and the public as team members in the research process. We encourage other myeloma researchers to do the same to ensure research is meaningful and relevant to the communities who rely on it.

**Supplementary Information:**

The online version contains supplementary material available at 10.1186/s40900-023-00476-9.

## Background

Over 160,000 people worldwide are currently living with multiple myeloma (commonly referred to as myeloma) [1], a rare cancer that affects the production of plasma cells and causes a multitude of symptoms, including reduced kidney function, bone lesions, and anemia [2]. Even though there is no known cure for this disease, myeloma patients are now living nearly three times longer than before [3, 4], thus highlighting the considerable need for researchers to begin to focus on topics deemed important to patients, caregivers, and healthcare providers to improve the lives of the patients and families affected by myeloma [5].

The James Lind Alliance is an organization focused on aligning research with the needs of patients, caregivers, and healthcare providers by developing a process for priority setting partnerships [6]. The aim of a priority setting partnership is to identify existing evidence gaps to ensure that funded health research is as relevant as possible to and most impactful for the communities that need it the most.

Recently, a pan-Canadian priority setting partnership was conducted to determine the research priorities of patients living with colorectal cancer [7]. The questions produced covered a range of topics that had not yet been fully addressed in the available literature, which emphasizes the key role that patients, caregivers, and clinicians play in ensuring that ongoing research is reflective of the priorities established by this community. A similar colorectal cancer-focused priority setting partnership was carried out in Germany, and parallel conclusions were drawn; patient-centred research is imperative to improving health research, and that collaborative partnership results in a rich “coproduction of knowledge” [8].

In the United Kingdom, a priority setting partnership was conducted to determine the priorities of young people diagnosed with cancer to inform research funding decisions and encourage further research with this community [9]. This population of patients, caregivers, and health professionals identified that research priorities should centre on holistic and psychosocial aspects of care delivery, in addition to traditional drug- and biology-focused research.

Furthermore, previous research has highlighted that, without the involvement of patient and public partners throughout the research process, results focus primarily on drug-centered treatments [10]. A survey of priority setting partnerships over a seven-year period found that patients and caregivers desired to learn about alternative treatments rather than traditional medical approaches to treating the disease under investigation [11]. This research highlights the discrepancy that exists between ongoing research and the needs of the respective communities.

The success of the priority setting partnership model can be attributed in large part to the involvement of patients, caregivers, and healthcare providers as equal partners in the decision-making process throughout the duration of the project. In doing so, this approach shifts the traditional research paradigm, thus allowing those who are directly affected by a health condition to take on an active role in seeking answers to their health-related questions [11].

### Purpose

The purpose of this research was to conduct a pan-Canadian James Lind Alliance priority setting partnership to inform future research about multiple myeloma by identifying the long-term research priorities in this field.

## Methods

### Context and scope

The priority setting partnership focused on adult patients living with multiple myeloma, their caregivers, and healthcare professionals. A steering group of patients, their caregivers, and relevant healthcare providers was formed to oversee every decision made for this project. From the outset, the steering group determined the scope of the project would encompass questions related to myeloma diagnosis, treatment, management, and living well with the disease. As a result, the project set out to identify the Top 10 unanswered questions related to these areas of myeloma research in Canada.

### Setting

Given the varying outcomes of this disease based on location alone [1, 12], the steering group felt that it was important to ensure that all patients, caregivers, and healthcare providers throughout the country were able to express their thoughts and opinions about their myeloma priorities. Therefore, the project was conducted nationally. We hosted 16 monthly meetings, most of these were held virtually using video conference software; the only exception was the second meeting, which was held in person in Toronto, Canada. This in-person meeting was an important step toward the initial decision-making of the project’s scope, identifying stakeholders, and creating communications plans. Between meetings, the research team maintained email communication with steering group members to share progress and ensure that there were no further topics to discuss.

### Governance and team

After learning of the James Lind Alliance and the process of priority setting partnerships, a medical oncologist (priority setting partnership lead) initiated the myeloma priority setting partnership with the help of the Maritime Strategy for Patient-Oriented Research (SPOR) Support for People and Patient Oriented Research and Trials (SUPPORT) Unit (MSSU), one of 11 units funded by the Canadian Institutes of Health Research established to support and promote Canada’s national Strategy for Patient-Oriented Research. This group will be hereafter referred to as the research team. The research team was responsible for the day-to-day operations of the myeloma priority setting partnership, including research activities (i.e., data analysis, evidence checks) and administrative tasks.

Shortly thereafter, a James Lind Alliance advisor was assigned to the newly established priority setting partnership to oversee the project and ensure that it was conducted based on the organization’s principals and procedures. Furthermore, the advisor served as an impartial facilitator who participated in all discussions and meetings for the duration of the project.

Finally, Myeloma Canada was secured as the project funder. Myeloma Canada is a non-profit, patient-led organization focused on funding research, advocating for patients, and educating newly diagnosed patients and caregivers on myeloma. In addition to funding the project, Myeloma Canada partnered with the research team and used their national network to identify potential steering group members. They also supported the implementation of the project by aiding in the dissemination of surveys, facilitating outreach, connecting with appropriate stakeholders, and advising on the research landscape.

Per the James Lind Alliance recommendations, neither the priority setting partnership lead, nor the research team had any voting rights or decision-making power throughout the course of the project. Likewise, while Myeloma Canada was a key partner in the priority setting partnership, its staff did not have voting or decision-making capacity.

### Stakeholders or participants

A significant component of this project was the establishment of a pan-Canadian steering group made up of patients, caregivers, and healthcare providers. While the priority setting partnership lead initiated the project, the steering group guided and oversaw all project planning and decision-making going forward. Patient and public partners were fully engaged in all aspects of the project as described below in accordance with the GRIPP2 form checklist (see Additional file 1).

Steering group members were identified through a process of peer-to-peer networking and snowball sampling. First, the priority setting partnership lead approached colleagues and known patients and caregivers with previous involvement in multiple myeloma advocacy to express interest in their involvement. Then, Myeloma Canada shared the engagement opportunity through their national network of support groups. From there, patients, caregivers, and healthcare providers expressed their interest in participating on the research team. These individuals also identified other peers and colleagues from across Canada who could potentially sit on the steering group.

The purpose of the project and its commitment level was explained to each individual expressing interest. Steering group members were selected based on a variety of criteria: level of interest and ability to commit, role (patient, caregiver, or healthcare provider, including specific healthcare profession), stage of disease (or of family member’s disease), stage of career, and location within Canada. These parameters were identified as having a potential impact on the experiences of people living with myeloma and were utilized to ensure as many voices as possible were amplified throughout the project.

The steering group consisted of nearly equal membership between patients (*n* = *3*), caregivers (*n* = *4*), and healthcare providers (*n* = *3),* to support inclusivity, diffuse power imbalances amongst members, and ensure that patients and caregivers were meaningfully involved. Of the ten members, three were from Atlantic Canada, four from Central Canada, one from the Prairie region, and two from the Pacific Region. To encourage and maintain an inclusive working environment, the preferred communication methods (i.e., email, phone call, video call) of each steering group member were noted and utilized.

To ensure that all decisions were being made by the steering group, the research team held monthly meetings to discuss project progress and provide group members the opportunity to ask questions and inform upcoming activities. Steering group members were encouraged to openly express their opinions on all topics. Regarding decision-making, the research team provided information and guidance, but ultimately, all decisions were taken following a vote by steering group members. At the end of each meeting, the James Lind Alliance Advisor gave each steering group member the unique opportunity to share their final thoughts. This was done to mitigate the effects of potential power dynamics between researchers and steering group members that may have prevented patients, caregivers, or healthcare providers from fully sharing their thoughts and opinions. Throughout the project, all meeting minutes (e.g., key discussion points, decisions, and future action items) were thoroughly documented and sent to the steering group for feedback and approval.

### Framework for priority setting

**Fig. 1:**
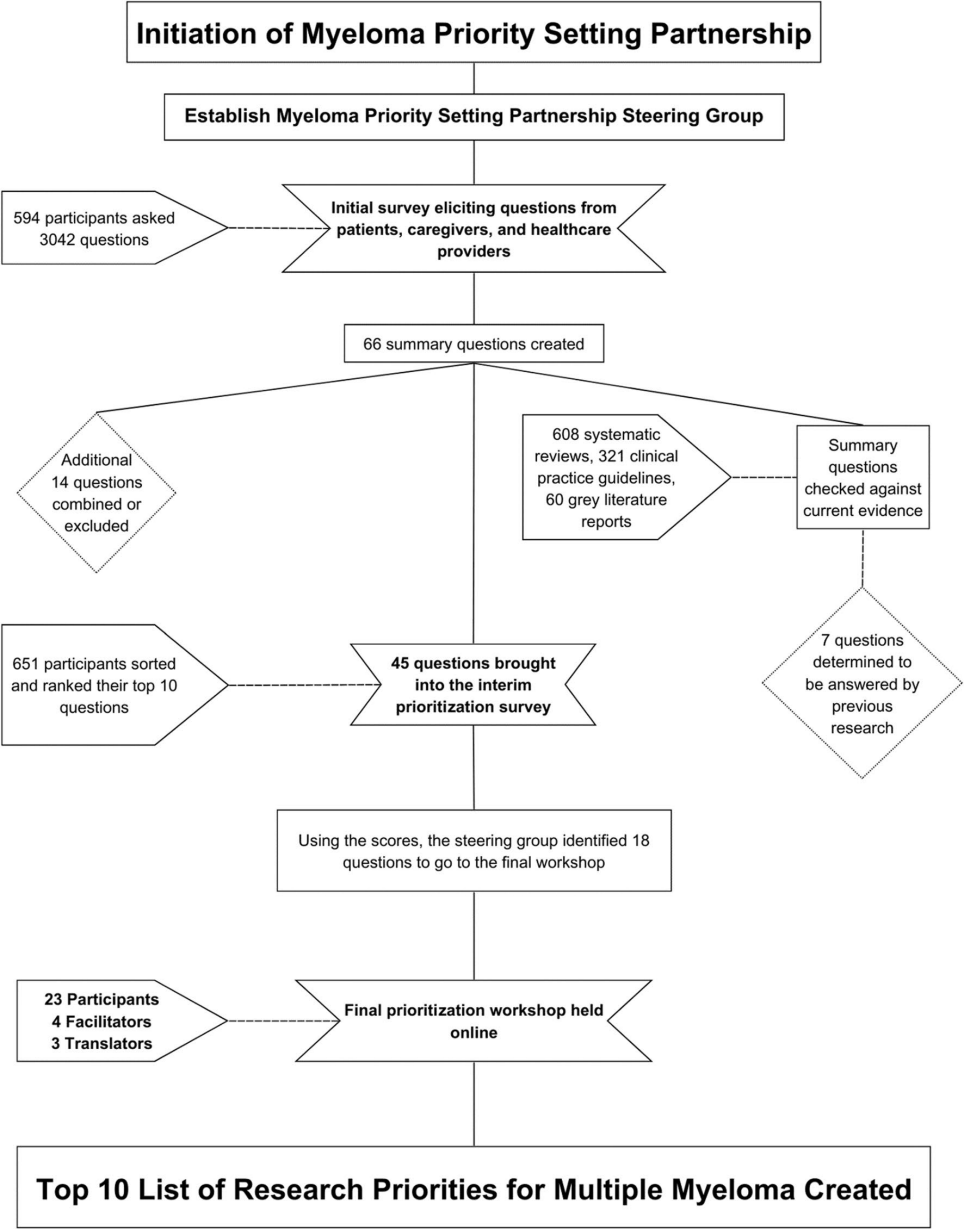
Flow chart of the Myeloma Priority Setting Partnership process

The myeloma priority setting partnership followed the iterative process developed by the James Lind Alliance (as visualized in Fig. 1) including an opening survey to gather questions from the myeloma community; an evidence check where questions were systematically verified to determine if they had been previously answered by research; a second survey to rank the unanswered questions; and a final priority setting workshop where the Top 10 questions were chosen and ranked using adapted nominal group techniques specific to the James Lind Alliance method [13], which were administered by trained James Lind Alliance facilitators.

### Identification and collection of research priorities

A member of the research team drafted the initial survey based on the recommendations of the steering group. The survey was reviewed by the steering group members and then distributed to others in their networks for pilot testing. The resulting survey included five open-ended questions to elicit participants’ questions about the four myeloma domains chosen as the scope for the project (i.e., diagnosis, treatment, management, and living well) and one question devoted to “other” queries. The survey ended with several demographic questions (e.g., age, ethnicity, disease status, province of residence, geography [urban or rural]) to ensure that all relevant voices were heard.

The first survey was open from October 2019 to January 2020, during which time it was distributed through Myeloma Canada’s email lists and social media accounts and through the networks of individual steering group members. The survey was conducted in English and French, both official languages of Canada, and was available in online and paper formats. The research team computed descriptive statistics for demographic variables and examined them regularly throughout the data collection phase to direct recruitment towards underrepresented groups (e.g., those living in rural areas). Per the recommendation of our James Lind Alliance advisor, these variables were used solely to guide recruitment efforts; we did not examine the relations between participants’ demographic characteristics and research questions. Unfortunately, since the demographic questions were placed at the end of the survey, incomplete surveys led to considerable missing data on these questions.

The survey generated 3042 questions from 594 participants (see Table 1), which exceeded the desired sample size of 500 participants, based on the size of the Canadian myeloma community and previously conducted priority setting partnerships. French survey responses were translated into English by a bilingual member of the research team before proceeding to the next phase.

**Table 1:** Demographic characteristics of initial survey participants

Demographic characteristic	
*Role*	
Person with myeloma	339 (57.07%)
Caregiver of person with myeloma	73 (12.29%)
Healthcare provider for people with myeloma	27 (4.55%)
Other^a^	15 (2.53%)
Not specified	140 (23.57%)
Total (*N*)	594 (100%)
*Language*	
English	481 (80.98%)
French	113 (19.02%)
Total (*N*)	594 (100%)
*Gender*	
Woman	271 (45.62%)
Man	184 (30.98%)
Prefer to self-identify	1 (0.17%)
Not specified	138 (23.23%)
Total (*N*)	594 (100%)
*Mean age *(*SD*)	63.50 (10.74)
*Race (note: participants could check all that applied)*	
Indigenous	5 (0.84%)
Arab or West Asian	3 (0.51%)
Black	3 (0.51%)
Chinese	3 (0.51%)
Filipino	1 (0.17%)
Japanese	1 (0.17%)
Korean	2 (0.34%)
Latin American	2 (0.34%)
South Asian	1 (0.17%)
South East Asian	5 (0.84%)
White	426 (71.72%)
Other	12 (2.02%)
Not Specified	138 (23.23%)
*Province*	
Alberta	50 (8.42%)
British Columbia	90 (15.15%)
Manitoba	12 (2.02%)
New Brunswick	20 (3.37%)
Newfoundland and Labrador	8 (1.35%)
Nova Scotia	20 (3.37%)
Ontario	161 (27.10%)
Prince Edward Island	4 (0.67%)
Quebec	80 (13.47%)
Saskatchewan	10 (1.68%)
Not Specified	139 (23.40%)
Total (*N*)	594 (100%)
*Community size*	
Extra Large Population Centre (more than 500,000)	148 (24.92%)
Large Population Centre (100,000–500,000)	118 (19.87%)
Medium Population Centre (30,000–100,000)	73 (12.29%)
Small Population Centre (1000–30,000)	80 (13.47%)
Rural area (less than 1000)	27 (4.55%)
Do not know	10 (1.68%)
Not specified	138 (23.23%)
Total ( *N*)	594 (100%)
*Mean years since diagnosis* (*SD;* *patient*)	5.58 (4.67)
*Current myeloma treatment (patient)*	
No	68 (20.06%)
Yes	258 (76.11%)
Not specified	13 (3.83%)
Patient sub-group total (*n*)	339 (100%)
*Mean years since diagnosis* (*SD;* *caregiver*)	5.07 (3.90)
*Healthcare profession*	
Physician	8 (29.63%)
Nurse	7 (25.93%)
Pharmacist	6 (22.22%)
Other	5 (18.52%)
Not Specified	1 (3.70%)
Healthcare provider sub-group total (*n*)	27 (100%)
*Mean years caring (SD; healthcare provider)*	12.96 (9.24)

^a^Others included bereaved caregivers, other family members, and people with related disorders

### Processing and verifying questions

Questions that did not fall into the scope of the project were identified and removed by the research team with approval from the steering group. The research team then reviewed and assigned codes to the remaining questions and grouped similar questions together into broad categories (e.g., side effects, maintenance medications, physical activity). The questions and corresponding codes were reviewed by the steering group. Once all of the questions were categorized, a summary question (known as an ‘indicative’ question) was developed by the steering group and research team to encompass all questions related to that code. This process produced a list of 66 indicative questions which were reviewed and approved by the steering group. More information about the indicative questions, including exemplary raw questions and related codes, can be found on the James Lind Alliance website (https://www.jla.nihr.ac.uk/priority-setting-partnerships/myeloma/top-10-priorities.htm).

These questions were brought forth to an evidence check to determine if they had been answered by prior research, notably systematic reviews and clinical practice guidelines. Given the relative scarcity of myeloma research and the breadth of questions, the research team used a broad search strategy to identify all such articles related to myeloma in the preceding ten years. Specifically, we searched the following databases for systematic reviews on multiple myeloma from 2010 until June 2020: MEDLINE All, Cochrane Database of Systematic Reviews, and Joanna Briggs Institute. Following this, we searched MEDLINE, International Myeloma Working Group, European Society of Medical Oncology, American Society for Medical Oncology, Advent Health Cancer Institute, National Institute of Health and Care Excellence, SPOR Evidence Alliance Clinical Practice Guidelines Database, and Google. Studies were excluded if they were conducted outside of the United Kingdom, United States, or Canada as these countries were determined to have similar disease treatment standards and outcomes. This search yielded 608 systematic reviews, 321 clinical practice guidelines, and 60 grey literature reports and articles.

Each article from the resulting search was screened by two members of the research team to first determine the general topic of the review or guideline, and then determine if it was relevant to the identified questions. From there, the research team compared the indicative questions to the existing evidence and made recommendations about whether it was answered by the prior research. The steering group reviewed the evidence and accepted these recommendations resulting in seven questions of the 66 being deemed answered by the available literature.

### Interim prioritization of research questions

Following the James Lind Alliance guidelines, the remaining questions were subjected to a second survey to generate initial rankings and select the questions for the future workshop; this is known as an interim prioritization survey. However, the steering group believed that 59 questions were too many given the time and/or cognitive difficulties experienced by patients from their disease. Therefore, they decided to reduce the number of questions by combining or excluding questions, a process guided by the steering group’s combined expertise. Ultimately, a total of 45 questions were included in this survey.

Available in French and English, the interim prioritization survey was conducted from November 2020 to December 2020 using Optimal Card Sort, a survey platform where participants could drag the questions they felt were important either into a ‘holding’ column or into their ‘top 10’ column and then rank them in order of importance from 1 (most important) to 10 (least important).

The survey platform offered a unique and interactive way to sort the questions and allowed us to present the 45 questions in random order to avoid bias towards questions as the top of the list. While participants could have ranked the Top 10 on paper, the experience and responses would not have been comparable to those who participated online. Therefore, the research team decided, with support from the steering group, to only offer the survey online. For this survey, the steering group and research team opted to collect only two pieces of demographic information: participants’ role (i.e., patient, caregiver, healthcare provider, other) and province of residence. These demographic questions were featured at the beginning of the questionnaire in order to minimize the risk of missing data. Participants were asked to respond to these questions and then choose their Top 10 questions and rank them from most to least important. At the end of the survey, participants were asked if they were interested in being considered for future phases of the study.

Any participant that had at least 10 cards sorted was included in the analysis for a total of 651 participants (see Table 2). The research team analyzed the responses to produce a score for each question based on how often it was included as a Top 10 question, and how high it ranked on participants’ Top 10 lists. This process was carried out separately for English and French surveys for patients, caregivers, healthcare providers, and total responses. As a result, each question was assigned four rankings for English responses, and four scores for French responses (see Additional file 2) because the linguistic groups vary geographically and may have different experiences with healthcare.

**Table 2:** Demographic characteristics of second survey participants

Demographic characteristic	
*Role*	
Person with myeloma	442 (67.90%)
Caregiver of person with myeloma	150 (23.04%)
Healthcare provider for people with myeloma	35 (5.38%)
Other^a^	22 (3.38%)
Not specified	2 (0.31%)
Total (*N*)	651 (100%)
*Language*	
English	559 (85.87%)
French	92 (14.13%)
Total (*N*)	651 (100%)
*Province*	
Alberta	68 (10.45%)
British Columbia	120 (18.43%)
Manitoba	25 (3.84%)
New Brunswick	24 (3.69%)
Newfoundland and Labrador	11 (1.70%)
Northwest Territories	1 (0.15%)
Nova Scotia	15 (2.30%)
Nunavut	1 (0.15%)
Ontario	256 (39.72%)
Prince Edward Island	2 (0.31%)
Quebec	110 (16.90%)
Saskatchewan	13 (2.00%)
Yukon	1 (0.15%)
Not Specified	4 (0.61%)
Total (*N*)	651 (100%)

^a^Others included bereaved caregivers, other family members, and people with related disorders

The research team discussed these scores with the steering group to determine which questions moved forward. The group chose to include the Top 15 rated questions overall as well as three questions that were ranked among the Top 5 by a single group (i.e., patient, caregiver, or healthcare provider) to ensure that all groups had their voices heard. This list of questions was then brought to the final workshop.

### Final prioritization of research questions

The final priority setting workshop was held in April 2021 and was designed to hold small group discussions to determine the Top 10 questions for future multiple myeloma research. Participants were contacted by the research team from the pool of interested survey participants and targeted recruitment through the steering group’s networks and asked to complete an expression of interest form. Expression of interest forms asked participants to identify their role (patient, caregiver, or healthcare provider), province of residence, gender identity, and racial and ethnic background. This information was used by the steering group and research team to select a diverse group of workshop participants (see Table 3). The desired sample size for the workshops was 24 participants, based on the James Lind Alliance guidelines for priority setting partnerships [13].

**Table 3:** Demographic characteristics of workshop participants

Demographic characteristic	
*Role*	
Person with myeloma	8 (34.78%)
Caregiver of person with myeloma	6 (26.09%)
Healthcare provider for people with myeloma	7 (30.44%)
Caregiver and healthcare provider	2 (8.70%)
Total (*N*)	23 (100%)
*Language*	
English	18 (78.26%)
French	5 (21.74%)
Total (*N*)	23 (100%)
*Gender*	
Woman	16 (69.57%)
Man	7 (30.43%)
Total (*N*)	23 (100%)
*Mean age (SD)*	53.29 (13.73)
*Race (note: participants could check all that applied)*	
South Asian	2 (8.70%)
Black	1 (4.35%)
Indian	1 (4.35%)
Arab or West Asian	3 (13.04%)
South East Asian	1 (4.35%)
Latin American	1 (4.35%)
White	15 (65.22%)
Not specified	1 (4.35%)
*Province*	
Alberta	2 (8.70%)
British Columbia	8 (34.78%)
New Brunswick	2 (8.70%)
Newfoundland and Labrador	1 (4.35%)
Ontario	4 (17.39%)
Quebec	6 (26.09%)
Total (*N*)	23 (100%)

Twenty-three individuals convened online through Zoom for two half-day sessions. All essential workshop materials were distributed to participants beforehand, both in virtual and hard-copy formats. During the workshop, James Lind Alliance facilitators led breakout groups in which attendees participated in small group discussions to share and explain their personal Top 10 list and engage in discussions to achieve group rankings. At the end of the first day, questions from each group were compiled in a list from 1 to 18 (highest to lowest priority).

On the second day of the workshop, new breakout groups were formed with the goal of determining a final list of questions ranked according to priority. Small groups decided on the Top 10, the next 11–14, and the final 15–18 questions. At the end of day two, the Top 10 lists from each group were combined again prior to a final discussion to ensure that this list was unanimously agreed upon.

Following the priority setting workshop, a final survey was administered to all participants to elicit final thoughts and input on the ranking of the questions, as well as feedback on the overall process.

## Ethics approval

This study was reviewed and approved by the Horizon Health Network Human Research Protection Program including the Research Ethics Board (RS#: 2019–2788). Informed consent was obtained from participants at each stage of the research process.

## Results

### Output

The project culminated in a ranked Top 10 list of research priorities for the future of myeloma research that is shared among people living with myeloma, caregivers, and healthcare providers (Table 4). This list encompassed questions related to all four areas of scope and highlighted research topics that may be underway but are still largely unanswered (i.e., finding a cure for myeloma). This list was previously published in a medical journal to raise awareness of these questions among myeloma clinicians, researchers, and funders [14].

**Table 4:** Top Ten Priorities for Multiple Myeloma Research

1	How can we cure myeloma?
2	Are novel immunotherapies effective for the treatment of myeloma?
3	How can we improve the diagnosis (e.g., faster, less invasive) of myeloma, and what is the impact of earlier diagnosis on patient outcomes?
4	What are new treatments for myeloma patients that will improve life expectancy with fewer adverse side effects?
5	How can we personalize a patient’s treatment based on their type of myeloma and genetic profile, and what is the impact of personalized medicine on treatment efficacy and disease outcomes?
6	How can we prevent bone deterioration and/or repair bones that have been damaged without negative side effects or surgery?
7	How can we safely reduce, cycle, or stop the use of anti-myeloma medications to reduce the side effects of treatment and maintain control over myeloma?
8	How can we reduce or manage the short- and long-term adverse effects of myeloma treatment?
9	What is the most effective way (i.e., drug combinations, sequence, frequency, and intensity) to treat refractory, relapsed, and drug resistant myeloma?
10	Can we develop treatments specifically for high risk or aggressive myeloma that will improve outcomes for these patients?

The remaining questions were also ranked from 11 to 18 and are available on the James Lind Alliance website (https://www.jla.nihr.ac.uk/priority-setting-partnerships/myeloma/top-10-priorities.htm).

### Evaluation and feedback

In all, we received feedback from 17 of the 23 participants (four patients, seven caregivers, and six healthcare providers). Participants were first asked to comment on the Top 10 and the Top 11–18 questions. Overall, participants were pleased with the order of the questions, regardless of whether their “personal” Top 10 questions were included in the final list:

[The Workshop] has been an important learning experience to better understand the next stages of the disease and its impacts on my life. I believe that the chosen order of priorities will have a positive impact for both newly diagnosed patients and for those who have been living with this disease and give hope for a cure, better quality of life, and longer life. (ID#2)

According to participants, small groups allowed for more in-depth discussions and were thus seen as an important component of the workshop. However, regarding the end of the second day, attendees would have preferred having more time during full-group discussions to understand more fully the final Top 10 list.

There were a few disagreements over the final ranking order as some participants felt that some questions should have been placed higher or lower on the list. At the same time, participants recognized that all 18 questions would be presented to myeloma researchers, and that some of the Top 10 questions, if answered, would help to answer the Top 11–18 ranked questions.

Despite these criticisms, participants valued their discussions with fellow workshop attendees, and felt hearing their stories allowed them to accept changing their personal Top 10 questions:

The questions I was less attached to were easy to let go of when I heard the perspectives of others and felt their emotions. Members on Day 1 and Day 2 greatly influenced how I prioritized most of the questions. I gained knowledge and perspectives that made it very easy for me to reprioritize the questions. (ID#4)

In general, participants felt that pre-workshop materials were helpful, as shown in Table 5. While 40% of participants found the paper-based materials extremely helpful, several participants indicated that mailing out a hard copy of the orientation package was not required and suggested asking future workshop participants to indicate their preference as a means of minimizing overall costs:

I would ask people if they need a paper version sent in the future as most people can print off a final version to work from. Saves time, money and the environment. (ID#10)

**Table 5:** Survey responses about pre-workshop material

	Not at all helpful	Not so helpful	Somewhat helpful	Very helpful	Extremely helpful	Total
Video presentation about the James Lind Alliance and the Workshop	0.00%	0.00%	25.00%	43.75%	31.25%	16
	0	0	4	7	5	
Workshop Information Pack	0.00%	0.00%	0.00%	37.50%	62.50%	16
	0	0	0	6	10	
Guidance on workshop technology and software	6.25%	0.00%	12.50%	37.50%	43.75%	16
	1	0	2	6	7	
Paper-Based Information Pack	6.67%	13.33%	33.33%	6.67%	40.00%	15
	1	2	5	1	6	

Participants were then asked about the flow of the workshop and how they felt about the large and small group sessions. Overall, everyone ‘Agreed’ or ‘Strongly Agreed*’* that these sessions were used effectively and that all attendees had the opportunity to openly share their thoughts and voice their opinions (Table 6). Most participants also ‘Agreed’ or ‘Strongly Agreed’ that the process of determining the Top 10 was robust and fair (Table 6). In the survey’s open-ended questions, participants said that they were pleased with how the workshop had been facilitated since everyone was able to voice their opinions while, at the same time, maintaining a set schedule:

The process could have been very overwhelming, conflictual, and challenging [;] however [,] by breaking into small groups [,] it was manageable and ensured everyone had opportunity to voice their thoughts. (ID#16)

**Table 6:** Survey responses on the priority setting workshop

How much do you agree or disagree that	Strongly disagree	Disagree	Neither agree nor disagree	Agree	Strongly agree	Total
Breaking up into small groups, and coming together again was an effective way to agree a Top 10 list of questions for research?	0.00%	0.00%	0.00%	31.25%	68.75%	16
	0	0	0	5	11	
The first large group session set the scene and provided information that helped me participate in the workshop	0.00%	0.00%	0.00%	50.00%	50.00%	16
	0	0	0	8	8	
I felt able to talk about my thoughts and opinions in the smaller group sessions	0.00%	0.00%	0.00%	12.50%	87.50%	16
	0	0	0	2	14	
In my small group sessions, I was able to keep track of the priority setting process	0.00%	0.00%	0.00%	12.50%	87.50%	16
	0	0	0	2	14	
Everyone was encouraged to join in with the discussions equally and had a chance to do that in the small group	0.00%	0.00%	0.00%	6.25%	93.75%	16
	0	0	0	1	15	
The final large group session provided an opportunity to review and agree the top 10 priorities for research	0.00%	0.00%	0.00%	37.50%	62.50%	16
	0	0	0	6	10	
The workshop facilitators were fair and independent	0.00%	0.00%	0.00%	6.25%	93.75%	16
	0	0	0	1	15	
The process of determining the Top 10 was robust and fair	0.00%	0.00%	6.25%	25.00%	68.75%	16
	0	0	1	4	11	

Finally, participants were asked if the workshop increased their knowledge of myeloma and priority setting in research. Many felt it did ‘a great deal’ or ‘a fair amount,’ while approximately one third of respondents did not feel the workshop increased their knowledge of myeloma (Table 7). The majority of respondents felt that the workshop provided an opportunity to learn from others ‘a fair amount’ or ‘a great deal,’ and many felt it provided an opportunity to inform others either ‘a great deal’ or ‘a fair amount’ (Table 7). In open-ended questions, participants expressed that, on a personal level, participating in the workshop proved to be a valuable experience:

The people in the groups I was in were all very supportive and encouraging. Everyone was respectful of others and listened to and asked questions relating to their experience or expertise. All participants were super engaged and eager to share their thoughts. It was such a great experience for me personally, even outside of the top 10 priority setting. (ID#1)

**Table 7:** Survey responses on what the workshop achieved

To what extent has your attendance at the workshop achieved the following?	Don’t know	Not at all	Not very much	A fair amount	A great deal	Total
Increased your knowledge about myeloma	0.00%	12.50%	18.75%	31.25%	37.50%	16
	0	2	3	5	6	
Increased your knowledge about setting priorities for research	0.00%	0.00%	0.00%	31.25%	68.75%	16
	0	0	0	5	11	
Provided an opportunity to learn from others	0.00%	0.00%	12.50%	25.00%	62.50%	16
	0	0	2	4	10	
Provided an opportunity to inform others	6.25%	0.00%	6.25%	37.50%	50.00%	16
	1	0	1	6	8	

Throughout the open-ended survey responses, participants offered suggestions to improve future consensus-building workshops. Among these were recommendations to facilitate a process for relationship building amongst participants, increasing the length of the two-day workshop by thirty minutes, and circulating the preliminary rankings between Day 1 and Day 2 so that participants could review and reflect on them on their own time.

The only thing I would say that if participants were open to being contacted by another participant, that there should be [a way] to do that. With an in-person meeting, that is easily done with the exchange of personal info, should anyone feel like they might want to connect at some point to share experiences etc. (ID#1)

Recommendations from survey participants were shared with the James Lind Alliance to improve future priority setting workshops.

## Discussion

The Canadian Myeloma Priority Setting Partnership was the first of its kind to bring together people living with myeloma, their caregivers, and healthcare providers involved in their care to identify the top priorities for future myeloma research. Our results demonstrate that among those involved, the priority is that current and future research aims to improve the treatment of this disease, including finding a cure.

Previous research conducted on the James Lind Alliance process demonstrated a discrepancy between the subject of disease-based research and the questions patients, caregivers, and healthcare providers want answered [10, 11]. In contrast to prior research which found that patients, caregivers, and healthcare providers favored questions related to holistic symptom management relative to pharmacological interventions [11], we identified Top 10 priorities that were largely related to pharmacological treatments, such as curative therapies and side-effect reduction. While this result was unexpected, we are confident that the results of the myeloma priority setting partnership accurately reflect the current needs of the myeloma community for a variety of reasons.

Workshop participants were satisfied with the Top 10 research questions and found the experience to be robust and enriching. Furthermore, a medical group from Oxford, United Kingdom, recently replicated our findings in their own local priority setting project, where seven of their Top 10 questions were related to drugs, treatments, and their side effects [15].

Questions related to living well (e.g., mental health and supportive care), lifestyle factors (e.g., physical activity and diet), and caregiver burden were identified as questions in the first survey, but following the interim prioritization surveys and final workshop, they were not ranked as highly. Thus, while these topics are perceived as worthwhile to the myeloma community; they pale in comparison to the research needed to improve myeloma treatment.

These results align with the current state of myeloma research. Over the past 30 years, the incidence of multiple myeloma has been rising steadily [3]; however, it was only in 2014 that the International Myeloma Working Group updated its diagnostic criteria to focus on biomarkers, and treatment for this disease could begin earlier and improve the life-expectancy of those diagnosed [2]. Given the lack of cure for myeloma, the emerging nature of treatments, and the side effects associated with them, it makes sense the myeloma community would prioritize research in these areas before non-pharmacological interventions or psychosocial needs. Nevertheless, it is worth noting that these questions were deemed unanswered by our evidence check and would be worthwhile exploring by researchers in these fields.

### Dissemination

After the consensus-building workshop and creation of the Top 10 list, the project entered its next phase, which involved disseminating the list as widely as possible to inform both the funding and the conduct of myeloma research. At this stage of the process, the steering group was dissolved, but members were still given the option to assist with knowledge translation activities. All patients and caregivers accepted to oversee the knowledge translation phase, while the healthcare providers regretfully withdrew their participation due to competing priorities and obligations.

The sub-group of interested steering group members (knowledge translation steering group) developed a knowledge translation plan based on target audiences, their preferred mode of research consumption, and associated costs. Knowledge translation steering group members were actively involved with knowledge translation, including informally sharing the results with peers (e.g., at support groups) and presenting to potential research funders while the research team developed traditional knowledge translation products (which were reviewed and approved by knowledge translation steering group members) tailored to the needs of the clinical and research members of the myeloma community. These knowledge translation products included a correspondence article in a peer-reviewed journal and presentations at national and international conferences [14].

Finally, the results were shared with Myeloma Canada who created a research grant to fund projects related to the identified priorities. In its inaugural year, Myeloma Canada committed $150,000 to two projects aligned with the results of the priority setting partnership [16].

### Knowledge gaps and future directions

While the present study highlights the current knowledge gaps in myeloma research that are pertinent to patients, caregivers, and healthcare providers, questions remain regarding the outcomes of the priority setting partnership approach. Currently, there is a lack of evidence that supports the identification of meaningful and practical outcomes of the priority setting partnership method beyond passive dissemination, such as the publication of findings in scientific journals [17, 18]. For this reason, future research investigating the applied products of priority setting partnerships, and specifically the myeloma priority setting partnership, should aim to identify what priorities have been addressed in practice, if further research is being carried out in these areas, and if top 10 priorities require updating as a result.

Many priority setting partnerships have found that patients ask for more research into holistic treatments and living well [11], but the myeloma priority setting partnership focused largely on pharmaceutical treatments and side effects. Research into new therapeutic agents for myeloma is ongoing and rapidly evolving. Therefore, it is important to consider that as the landscape of myeloma treatments and research changes, so will the priorities of those affected by the disease. Currently, there are no guidelines on how often updates to priorities should occur, but it is important to consider revisiting priorities to determine when they have been answered by research [21].

Success of this priority setting partnership will be defined by research funding directed to the priorities identified through this initiative and the amount of original research, systematic reviews, and clinical practice guidelines aimed at addressing these questions. Ultimately, when these questions have been answered by a rigorous evidence check, such as the one performed during this project, the myeloma priority setting partnership will have achieved its goal.

Furthermore, given that one aim of the myeloma priority setting partnership is to inform the funding of research related to the identified priorities, grant mapping would be an appropriate tool to measure the long-term impact of the priority setting partnership [16, 19]. This process involves identifying links between Top 10 priorities and relevant journal abstracts and tracking the number of citations generated from our myeloma priority setting partnership. Moreover, Myeloma Canada will use the insights garnered from our myeloma priority setting partnership to identify and share their funding, research, and fundraising priorities.

These priorities can also be used to apply to other research fundings organizations such as the Canadian Cancer Society and Canadian Institutes of Health Research. These organizations emphasize a person-centred approach to cancer prevention, detection, and care, and recognize the value of engaging patients at all stages of the research process. Moreover, the priorities identified through this project align with their strategic goals. For example, the Canadian Cancer Society strategic plan includes the need to develop new and more precise therapies to extend cancer survival and improve quality of life during and after treatment [20, 21].

### Lessons learned from patient engagement

Patient and public engagement was an integral part of this project, and without it, our results would likely not meet the needs of knowledge users in the myeloma and research communities. This engagement allowed for the formation of precise, indicative questions that ultimately led to a rich discussion at the final workshop, and results that are meaningful to the community at the focal point of the research. As with any research project involving patient engagement, there were some lessons that are important to highlight for future research.

First, it is imperative to ensure patients and caregivers understand the process of a priority setting partnership in its entirety before agreeing to join a steering group. To avoid potential barriers to participant involvement, transparency around the expected timeline and level of commitment required for involvement is paramount, with the understanding that completion could take longer than initially expected [22].

Second, initial informal meetings held for steering group members were beneficial for relationship development at the onset of the project and allowed members to gauge compatibility with other group members. Although the subsequent meetings were held virtually due to the COVID-19 pandemic, we feel that, when possible, future priority setting partnerships should aim to hold some steering group meetings in-person to fully engage patient partners and build cohesiveness between the research team and patient and public partners.

Third, frequent contact with patient and public partners in between meetings was valued and improved communication throughout the duration of the project. This ensured that patients were aware of project details, were involved in the legwork of the project, had ample opportunities to voice their concerns, and felt like valued members of the research team.

### Limitations

The present study used a robust, transparent, and multi-stakeholder approach to identify the priorities of myeloma research stakeholders; however, some limitations to this process exist. Our primary method of survey distribution was via channels of communication that are frequently utilized by Myeloma Canada and affiliate organizations. While there was a great uptake of survey completion, our sample was mostly comprised of active members within the myeloma community. As a result, the views expressed by the study participants may not be representative of the myeloma community in its entirety, and research priorities for individuals who do not receive frequent communications about myeloma may differ from those who do.

Furthermore, steering group members were comparable demographically; most resided in large metropolitan centres and identified similarly across racial and ethnic groups. Future research should aim to prioritize diversity across the steering group and study participants to ensure that myeloma research priorities are consistent across demographic groups and a variety of voices are amplified during the research process.

We experienced challenges recruiting healthcare providers as study participants. We conducted targeted outreach to professional organizations including: Canadian Clinical Trials Group, Canadian Medical Association, Canadian Association of Pharmacy in Oncology, Myeloma Canada Research Network, Canadian Association of Nurses in Oncology, Cell Therapy Transplant Canada, Canadian Hematology Society, Canadian Society of Hospital Pharmacists, Canadian Association of Pharmacy Technicians, Neighbourhood Pharmacy Association of Canada, Canadian Association for Population Therapeutics, Canadian Association of Medical Oncologists, Canadian Hematology Society, all Provincial and Territorial Medical Associations, all Provincial and Territorial Pharmacy Associations, and all Provincial and Territorial Health Authorities. Despite our best efforts, the proportion of healthcare provider respondents relative to patients and caregivers remained low, which could be due to the generally low response rates of healthcare providers and the length of our survey [23]. However, we ensured that the healthcare provider perspective was equally represented on the steering group and at the final priority setting workshop.

The onset of the COVID-19 pandemic during the project precluded in-person meetings of the steering group (aside from the initial “kick off” meeting) and the consensus-building workshop. Most priority setting workshops are held in person and allow for a greater degree of collaboration and networking than virtual workshops, while encouraging participants to be fully immersed in the priority setting experience [24]. Nevertheless, holding our priority setting workshop virtually also led to some advantages, such as less necessary travel time for participants (even if the costs were covered), eliminating the need for alternative caregivers, and avoiding putting immunocompromised patients at risk. The James Lind Alliance adjusted the workshops into virtual sessions and thus enabled us to reach a more diverse group of individuals who may not have possessed the capacity or health to travel.

The pandemic also contributed to lost momentum when it came to support from regional health authorities and organizations as their focus was understandably diverted to COVID-19. The pace of the project was subsequently reduced out of respect for the team’s mental health during lockdown. This ensured they had ample time for self-care and avoided becoming overwhelmed from project-related tasks. Taken together, these factors led to the project taking two years as opposed to the 12–18 months recommended by the James Lind Alliance [13].

## Conclusion

This project highlights important future directions for research on multiple myeloma and garners insight into the priorities of people living with this disease, such as the improvement of symptoms, its impact on daily life, and how medical research can work to improve the lifespan of myeloma patients. To further disseminate the Top 10 Priorities for Multiple Myeloma Research, we intend to follow the direction of previous James Lind Alliance priority setting partnerships [25–28] by using our generated list to encourage government agencies and foundations to fund research in these priority areas. Targeting Top 10 priorities will ultimately improve patients’ burden of disease, their quality of life, and symptom management. This project also underscores the value of patient and public engagement in research; the priority setting partnership approach results in participatory research that is meaningful and relevant to the communities that are affected by multiple myeloma. In this way, we can begin to close the knowledge-to-practice gap and improve the lives of people living with this disease.

## Supplementary Information

**Additional file 1**. GRIPP-2 Check List.

**Additional file 2**. Interim Prioritization Survey Results.

## Data Availability

Data and materials from this project, including questions, combined indicative questions, and evidence related to the questions is publicly available on the James Lind Alliance website (https://www.jla.nihr.ac.uk/priority-setting-partnerships/myeloma/).
