# Supplementary material for: How should multiple myeloma research change in a patient-oriented world? Findings and lessons from the pan-Canadian myeloma priority setting partnership
Source: Res Involv Engagem. 2023 Jul 29;9:60. doi: 10.1186/s40900-023-00476-9 (PMC10386308; doi:10.1186/s40900-023-00476-9)
Supplement: Supplementary file 1 — Additional file 1. GRIPP-2 Check List. [file 40900_2023_476_MOESM1_ESM.docx]

Additional File 1. GRIPP-2 Check List

| **Section and topic** | **Item** | **Reported on page No** |
| --- | --- | --- |
| 1: Aim | Report the aim of PPI in the study | 3 |
| 2: Methods | Provide a clear description of the methods used for PPI in the study | 3-4 |
| 3: Study results | Outcomes—Report the results of PPI in the study, including both positive and negative outcomes | 12-13 |
| 4: Discussion and conclusions | Outcomes—Comment on the extent to which PPI influenced the study overall. Describe positive and negative effects | 12 |
| 5: Reflections/critical perspective | Comment critically on the study, reflecting on the things that went well and those that did not, so others can learn from this experience | 12-13 |
